# Breeding maize (*Zea mays*) for Striga resistance: Past, current and prospects in sub‐saharan africa

**DOI:** 10.1111/pbr.12896

**Published:** 2021-03-01

**Authors:** Abdoul‐Madjidou Yacoubou, Nouhoun Zoumarou Wallis, Abebe Menkir, Valerien A. Zinsou, Alexis Onzo, Ana Luísa Garcia‐Oliveira, Silvestro Meseka, Mengesha Wende, Melaku Gedil, Paterne Agre

**Affiliations:** ^1^ Laboratoire de Phytotechnie, d’Amélioration et de Protection des Plantes (LaPAPP) Département des Sciences et Techniques de Production Végétale (STPV) Faculté d’Agronomie Université de Parakou Parakou Bénin; ^2^ International Institute of Tropical Agriculture (IITA) Oyo Road PMB 5320 Ibadan Nigeria; ^3^ Institut National des Recherches Agricoles du Bénin 01 BP 884 Cotonou Bénin; ^4^ Excellence in Breeding (EiB) CIMMYT ICRAF House UN Avenue PO Box 1041‐00621 Nairobi Kenya

**Keywords:** breeding strategies, maize, QTL, resistance, Striga

## Abstract

*Striga hermonthica*, causes up to 100% yield loss in maize production in Sub‐Saharan Africa. Developing Striga‐resistant maize cultivars could be a major component of integrated Striga management strategies. This paper presents a comprehensive overview of maize breeding activities related to Striga resistance and its management. Scientific surveys have revealed that conventional breeding strategies have been used more than molecular breeding strategies in maize improvement for Striga resistance. Striga resistance genes are still under study in the International Institute for Tropical Agriculture (IITA) maize breeding programme. There is also a need to discover QTL and molecular markers associated with such genes to improve Striga resistance in maize. Marker Assistance Breeding is expected to increase maize breeding efficiency with complex traits such as resistance towards Striga because of the complex nature of the host‐parasite relationship and its intersection with other environmental factors. Conventional alongside molecular tools and technical controls are promising methods to effectively assess Striga in Sub‐Saharan Africa.

## 1 INTRODUCTION

Maize is one of the most important cereal crops grown worldwide. In Sub‐Saharan Africa (SSA), it is regarded as the most important staple crop with huge potential for addressing the challenge of food insecurity (Abdoulaye et al., 2018). However, its productivity remains relatively low across SSA countries when comparing to the global average production (FAO, 2018). Amongst the major constraints that affect maize productivity, drought, low fertility and the parasitic weeds known as *Striga hermonthica*, have been recognized by farmers as the most widespread stresses (Atera et al., 2013; Edmeades, 2013; Das et al., 2019).

Striga, is a parasitic weed belonging to the Orobanchaceae family. It infests and reduces yields of many cereal crops including maize by up to 100% (Atera et al., 2013; Chemisquy et al., 2010; Parker, 2012; Teka, 2014). Across the globe, more than 50 species belonging to the Orobanchaceae family are identified and known as crop pests. In SSA, *S. hermonthica* (Del.) Benth. and *S. asiatica* (L.) Kuntze are the most economically important species affecting maize production (Menkir et al., 2012; Teka, 2014). According to Parker (2012), the tropical semi‐arid climatic conditions have allowed rapid development of the Striga and even its adaptation to context. Unfortunately, *S. hermonthica* infestation appears to be worsening due to the current intensive land use, mono‐cropping practices and human demographic pressure. All these factors lead to a continuous decline in soil fertility, which greatly favours the Striga occurrence (Rich & Ejeta, 2008). In West Africa, Striga is widely found across the region where maize yield losses due to infestation can vary from 20% to 80% ( Ejeta, 2007; Kim et al., 2002).

In the last few decades, efforts have been made to develop methods for Striga control, including agronomic cultural practice, biological control, chemical, host plant resistance and genetically modified crops. However, these strategies are only moderately effective, because Striga are still expanding its natural range by causing more yield losses. From existing strategies, the most effective and sustainable control seems to be an integrated approach that uses resistant cultivars (Chitagu et al., 2014; Hearne, 2009; Yoder & Scholes, 2010). Striga‐resistant maize can be a major component of integrated control if resistance is incorporated into adapted and, regionally productive cultivars. Resistant maize cultivars can, indeed, reduce both new Striga seed production and the Striga seed bank in infested soils. Significant progress towards the development of Striga‐resistant maize varieties have been achieved around the world, particularly in Africa. The International Institute of Tropical Agriculture (IITA), Ibadan, Nigeria and International Maize and Wheat Improvement Centre (CIMMYT), Zimbabwe, have developed several maize genotypes with varied Striga resistance levels and adapted to different eco‐climatic conditions. Yet, very few of these varieties are effectively resistant to Striga, because, they are continuously tolerant to the emergence of Striga plants. Thus, adding each year more Striga seeds into the soil after each growing season. Therefore, additional genes or sources of Striga resistance need to be found for introgression into maize elite varieties in order to develop varieties that support little or no Striga emergence. This review intends to give a brief update on current work towards Striga resistance emphasizing breeding methods for Striga resistance in Africa and the use of integrated Striga control mechanisms on maize.

## 2 ECONOMIC IMPACT OF Striga INFESTATION ON MAIZE PRODUCTION AND BIOLOGY OF *Striga* spp.

### 2.1 Economic impact of Striga infestation on maize production

**FIGURE 1:**
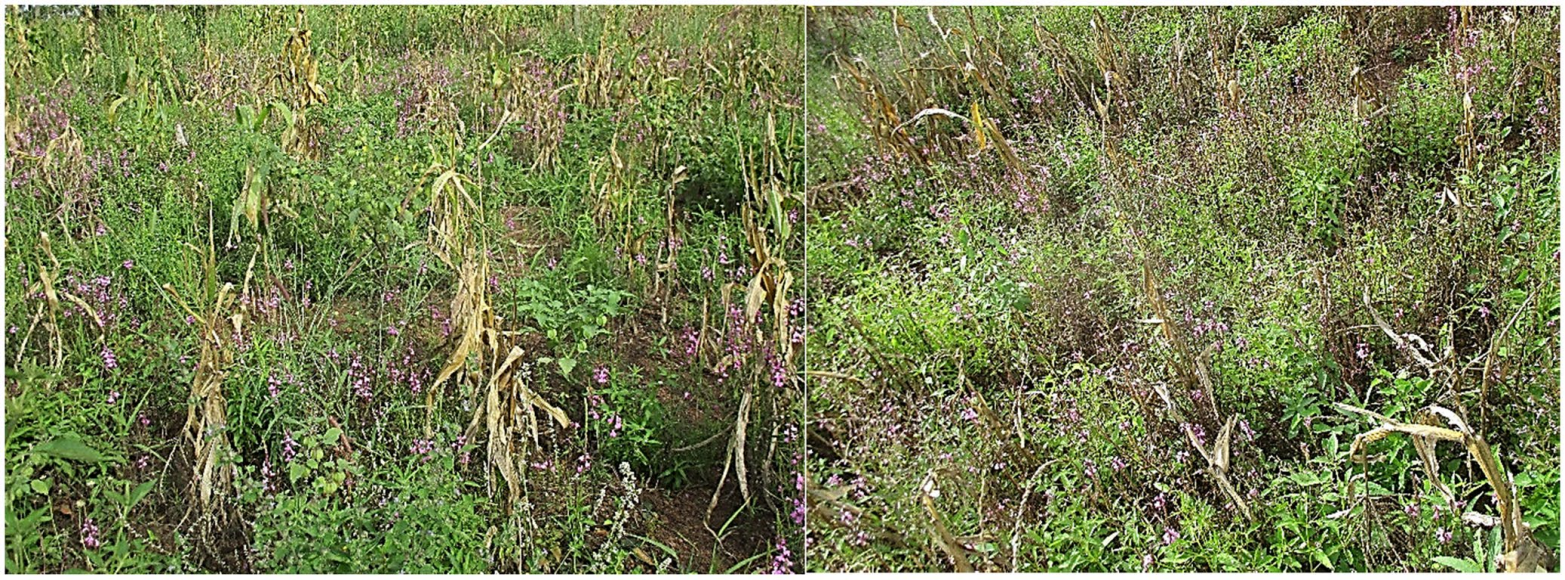
Maize field devastated by *S. hermonthica* in the North of Benin Republic *Source*: Yacoubou (2018)

Striga parasitism is a limiting factor to maize (*Zea mays* L.) cropping in the savannah zones of Sub‐Saharan Africa (SSA) which constitutes the maize belt of the sub‐region (Runo & Kuria, 2018). About 75% of cultivated land with maize in SSA is endemic to *S. hermonthica* (Akaogu et al., 2019). Maize yield losses under severe Striga infestation can be as high as 100% (Figure 1) and are economically estimated to $7 billion in the SSA alone (Spallek et al., 2013). The Striga problem has been worsened by the increasing mono‐cropping practice instead of rotation and intercropping systems, human demographic pressure on available land where up to 300 million farmers were exposed to the Striga infestation in SSA (Badu‐Apraku & Fakorede, 2017). Challenges in managing Striga infestation lead to agricultural land abandonment in several West African countries including Benin, Burkina Faso, Niger, Nigeria and Togo (Atera & Itoh, 2011; Badu‐Apraku, 2010; Badu‐Apraku et al., 2014). Consequently, this has threatened food security and livelihoods of millions farmers in most countries in this region (Menkir et al., 2020).

### 2.2 Biology and Striga spp. life cycle

**FIGURE 2:**
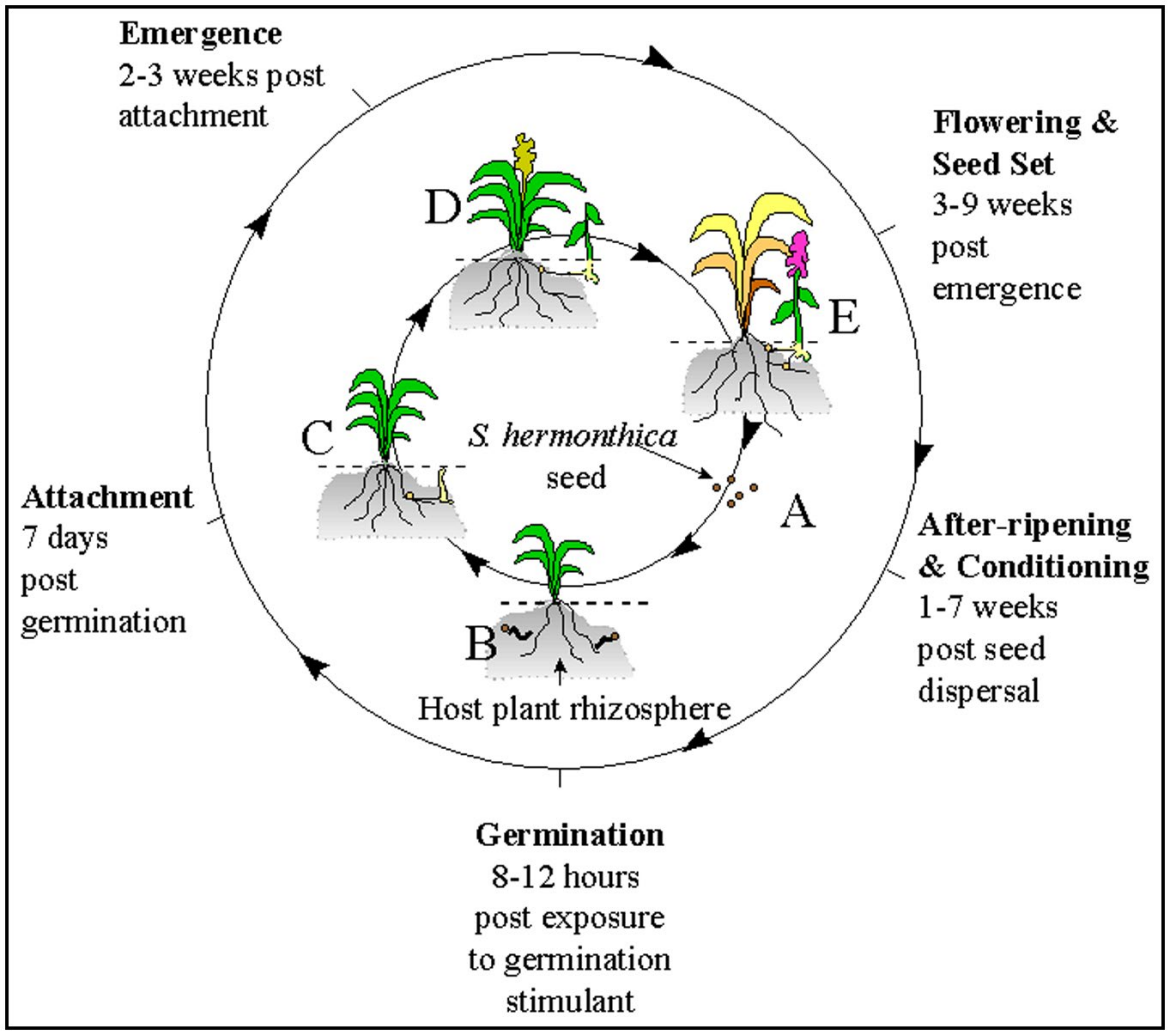
The life cycle of *S. hermonthica* on a susceptible host. Stages indicated: A = after‐ripening and conditioning of *S. hermonthica* seed, B = germination of *S. hermonthica* seed, C = haustorial initiation and attachment of *S. hermonthica* to the host followed by a period of growth underground, D = emergence of *S. hermonthica* plants from the soil, E = flowering, insect pollination, seed set and dispersal. Duration of each phase of the life cycle is indicated. *Source*: Hearne (2001) [Colour figure can be viewed at wileyonlinelibrary.com]

**FIGURE 3:**
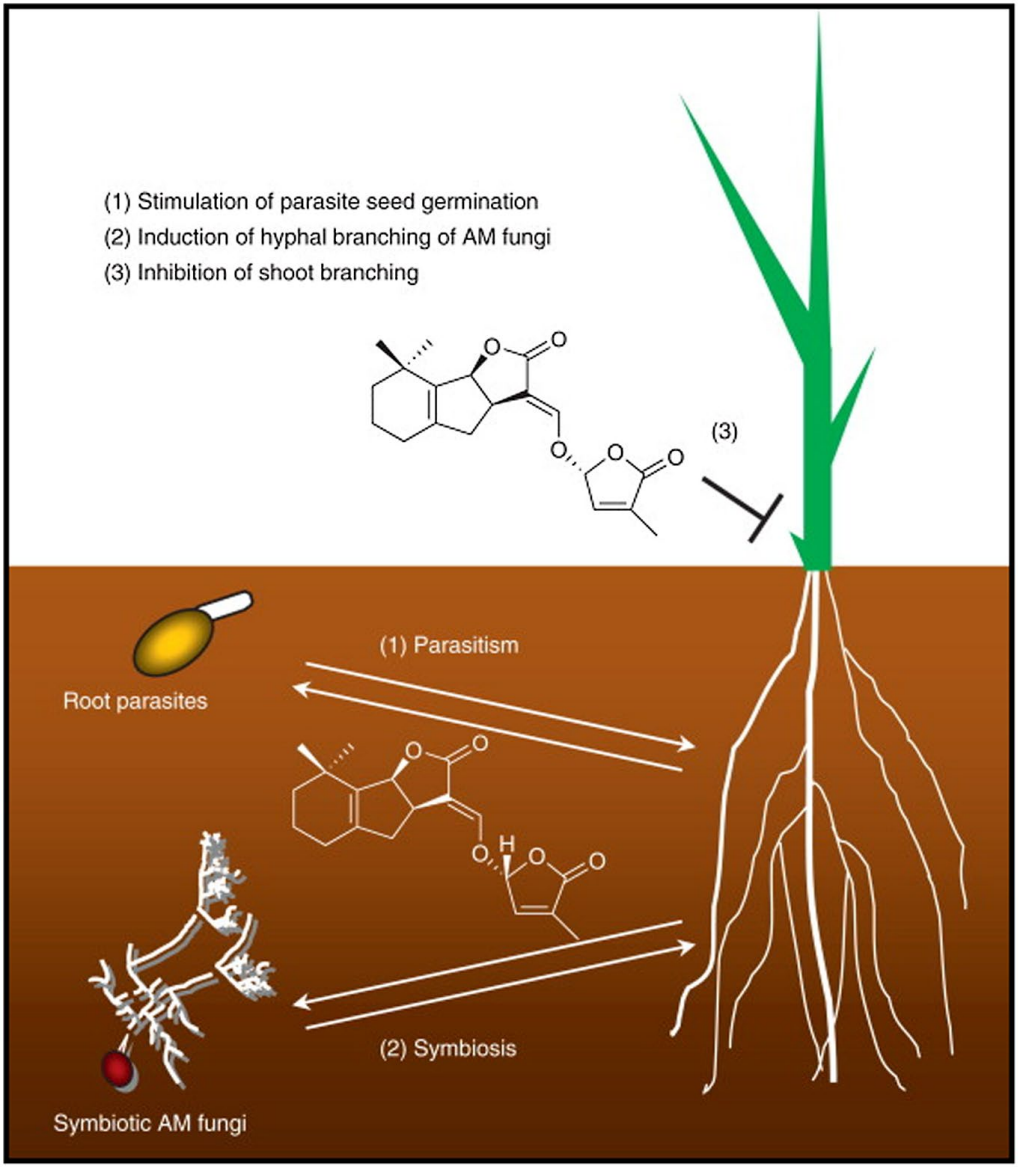
Biological functions of strigolactones *Source*: Yamaguchi et al. (2010) [Colour figure can be viewed at wileyonlinelibrary.com]

Life cycle of Striga is synchronized to that of its host and involves mechanisms that coordinate lifecycles of both the parasite and the host (Bouwmeester et al., 2003). Striga life cycle generally involves: germination, host attachment, formation of haustoria, penetration and establishment of vascular connections, nutrients accumulation, flowering and seed production (Parker & Riches, 1993) (Figure 2). Germination of Striga seeds depends on the presence of hormones known as strigolactones that are produced by the host and in other cases non‐host species (Spallek et al., 2013). With the presence of strigolactones, parasite seedlings attach to the host and form vascular connections depriving it of its water, carbohydrates and minerals (Yoshida & Shirasu, 2009). Under stressful conditions plant roots exude strigolactone hormone to promote symbiotic relationship with soil microbes for mineral nutrient scavenging (Steven, 2014). Parasitic plants such as *Striga hermonthica* have exploited these strigolactone hormones as signals to stimulate the germination of their seeds (Runo et al., 2012) (Figure 3). During early stages of seed development, before emergence, the parasite depends totally on the host plant (Webb & Smith, 1996). At this stage of subterranean development, *S. hermonthica* inflicts maximum damage to the maize plant. The adverse effect of Striga on maize is manifested as stunting, chlorotic and necrotic lesions on the leaves and reduction of ear size and grain yield (Adetimirin et al., 2000). Striga spp. take about 4–10 weeks to complete its life cycle after emergence and this completion usually occurs after harvest of the host (Ramaiah et al., 1983).

### 2.3 Striga control methods

Striga control is essential to ensure food security in the SSA (Ejeta, 2007; Rodenburg et al., 2005). Several methods, ranging from agricultural practices to biological control exist and significant progress has been made in Striga control research within Africa (Table 1).

**TABLE 1:** Striga management methods used in African countries

Methods	Factors in favour of control options	Setbacks for control options	References
Manual weeding	Reduction of *Striga* seed bank, easy to implement	Yield benefit is not immediate, labour intensive	Babiker (2007), Ayongwa et al. (2010 **)**
Crop rotation	Increase soil fertility, reduction of *Striga* seed bank	Benefit accruement requires time, costly as per family food	Carsky et al.(2000), Manyong et al. (2008)
Hand pulling	Reduction of Striga seed bank if performed before flowering, increase in yield	Inappropriate disposal increases seed bank	Jamil et al. (2011), Oswald (2005)
Push and pull	Provide livestock feed, reduction of *Striga* seed bank, control of stem borer, improvement of soil fertility	Costly to implement initially, benefit accruement requires time, trap crop used uneconomical	Khan et al. (2010), Hailu et al. (2018)
Fertilizer Application (N and P)	Increase in yield, improvement of soil fertility, reduction of *Striga* incidence	Costly to implement, labour Intensive	Jamil et al. (2012)
Intercropping with Legumes	Reduction of *Striga* seed bank, increase soil fertility, provide additional income	Labour intensive, trap crop used uneconomical	Bilalis et al. (2010), Ibrahim et al. (2014), Hailu et al. (2018)
Seed dressing (herbicide)	Increase in yield, easy to implement, Reduction of *Striga* incidence	Purchase of seed every season is costly May not be easy to implement	De Groote et al. (2008), Kanampiu et al.(2003)
Compost application	Increase in yield, easy to implement, reduction of Striga incidence, increase soil fertility	Increase pests, labour intensive	Osman et al. (2013)
Resistant Varieties	Easy to implement, high crop yield	Purchase of seed every season is costly, gene recombination's in the parasite (mutation), limited of resistance sources	Kouakou (2014), Naitormmbaide et al. (2015)
Herbicide Application	Reduction of Striga seed bank	Unavailable to farmers, cost prohibitive	Hesammi (2013); Ransom et al. (2012)
Biocontrol agent	Reduction of *Striga* emergence, improvement of crop yield Reduction of Striga incidence, increase yield, provide livestock fed	Labour intensive, source limited Crop uneconomical to farmers without livestock	Khan et al. (2010), Nzioki et al. (2016), Kountche et al. (2019)
Integrated approach: biocontrol agent and resistant varieties	Suppressing emergence and fecundity, germination and photosynthetic inhibition	Failure of the host's rhizosphere to maintain enough pathogen levels that guarantee control of the weed.	Ouédraogo et al. (2018), Shayanowako et al.(2018), Zarafi et al. (2015)
Integrated Striga Management: agronomic practices and resistant varieties	Reduction Striga emergence, reduction Striga infection levels and seed numbers in the soil, Increase in yield	Low adoption of these varieties, purchase of seed every season is costly, unavailability of resistant varieties to *Striga* species attacks, Mutation or geographical changes that occur over a number of years	Randrianjafizanaka et al. ( 2018), Ronald et al. (2019), Schut et al. (2015)

Cultural practices such as manual weeding, push and pull, crop rotation with non‐host intercrops (trap crops), fertilizer application, soil and water management, and transplanting have been attempted, but they offered limited success in controlling Striga infestation (Oswald & Ransom, 2002; Fasil & Verkleij, 2007; Udom et al., 2007; Manyong et al., 2008; Ayongwa et al., 2010; Lagoke & Isah, 2010; Hailu et al., 2018). Inter‐cropping cereals with legumes is another low‐cost and viable strategy that has been reported to influence Striga spp. infestation (Carsky et al., 2000; Akanvou et al., 2006; Kanampiu et al., 2018). Legumes, through their roots, fix atmospheric nitrogen, add organic matter to the soil by contributing to soil conservation, preserving the streamline soil moisture and enhances soil biodiversity, thereby improving soil health and fertility, which directly contributes to Striga control. Intercropping legumes with cereals reduces *S. hermonthica* but does not eliminate the parasite (Khan et al., 2000, 2007).

Other methods for Striga control include biological control using herbicide‐resistant maize variety (Imazapyr treatment), development of Striga‐resistant germplasm, use of fungus Fusarium isolation by applying strigolactones (Kanampiu et al., 2002; Ejeta, 2007; Illa et al., 2010; Nzioki et al., 2016; Uraguchi et al., 2018; Zwanenburg, & Blanco‐Ania, 2018; Kountche et al., 2019). All these approaches have been used with some degree of success to minimize the effect of Striga in maize production. The mode of action for each approach is different. For example, in the case of fungus, when *F. oxysporum* gets in contact with maize plants, there is a production of amino acids (L‐leucine and L‐tyrosine), that disrupt plant growth and development. These amino acids are toxic to Striga plants but innocuous to maize plants (Nzioki et al., 2016). The use of this biological control tool allowed the increment of more than 45% maize yield in Striga endemic zones in Kenya (Nzioki et al., 2016). Strigolactones (SLs) reduce the accumulation of abscisic acid (ABA) in plant by up‐regulating the ABA catabolic enzyme gene CYP707A1 (Lechat et al., 2015; Toh et al., 2012). The ABA is released by maize infected with *S. hermonthica*, that subsequently trigger stomatal closure to minimize water loss. SLs also increase the production of gibberellins (GA) hormones by up‐regulating gibberellin3β‐dioxygenase 1, which is involved in GA biosynthesis (Toh et al., 2015; Yao et al., 2016). Although ABA and GA represent central plant hormones and are known to antagonistically regulate seed germination in non‐parasitic plants, the effects of their exogenous application vary across parasitic plant species. Zehhar et al. (2002) and Toh et al. (2015), reported that neither GA nor ABA alone is sufficient to stimulate or inhibit seed germination in *S. hermonthica,* while Kannan and Zwanenburg (2014) and Zwanenburg et al. (2016) reported SLs application appears attractive owing to their decomposition in the soil within a short period. Nevertheless, the use of natural SLs for decomposition in soil does not seem a realistic alternative because the synthesis of these compounds is very labourious. More recently, genetic engineering has offered the promise of rapidly achieving resistance against Striga spp. Recent findings have shown that RNAs freely translocate between parasitic plants and their hosts (Kim & Westwood, 2015). This translocation suggests a possibility that RNA‐interference (RNAi) could be used as a potential tool to interfere in vital processes within the parasite by transforming the host with an RNAi construct that targets gene sequences specific to the parasite (Shayanowako et al., 2017). This technique is constrained by the lack of genes to target for silencing as well as by the delivery of iRNAs into the parasite (Kirigia et al., 2014). This constrain can be overcome using viral induced gene silencing (VIGS). Using a *Tobacco Rattle Virus* (TRV) – VIGS system, Kirigia et al. (2014) have shown that this system works in *S. hermonthica* and has been proven as a useful system for candidate gene validation either in parasite development or parasitism, for the development of resistant transgenic maize.

## 3 GENETICS RESISTANCE MECHANISMS TO Striga IN MAIZE

### 3.1 Resistance mechanism to Striga in maize

**FIGURE 4:**
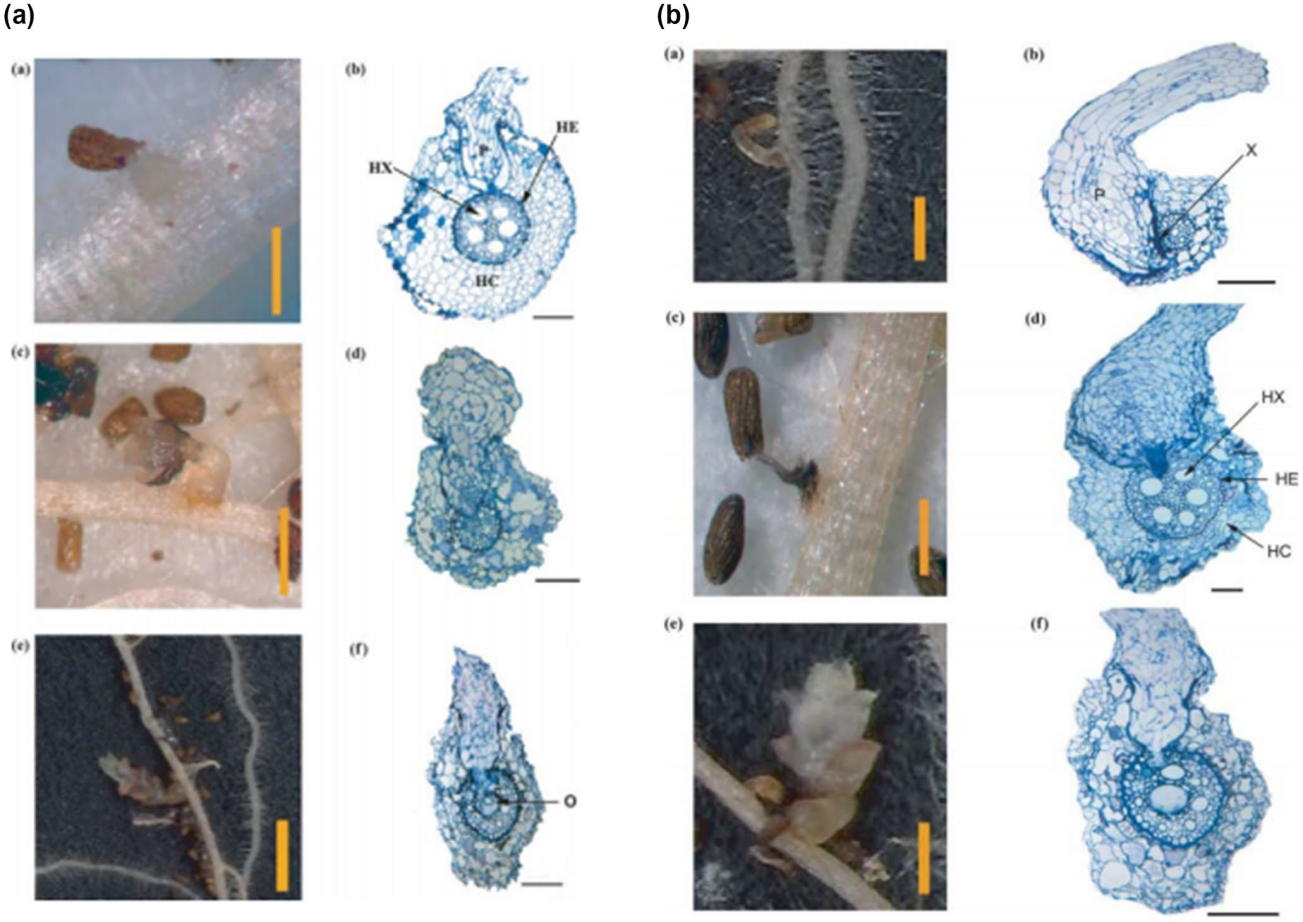
Mechanisms of resistance to *S. hermonthica* in maize *Source*: Amusan et al. (2008)

Striga resistance mechanisms act either before (preattachment) or after physical contact with the host (postattachment). Preattachment resistance (Figure 4a) occurs when a host produces low amounts of strigolactones or when Striga receptors that perceive germination stimulants are insensitive to the strigolactone levels produced by the host (Lumba et al., 2017; Mutinda, 2018). Binding causes the degradation of an F‐box protein, which in turn activates gene regulatory processes that lead to Striga germination (Lumba et al., 2017). It can also be due to the production of low haustorial initiation factors whose effect leads to a failure by Striga to develop haustorium effectively (Rich et al., 2004). Crop genotypes with preattachment resistance mechanism produce relatively low SLs, thereby inducing the germination of less parasitic seeds and consequently prevent the host plant from parasitism. Preattachment resistance has been shown in 'KSTP’94', an open‐pollinated maize variety used by farmers in Eastern Africa for *S. hermonthica* management. This maize variety was shown to produce low amounts of sorgomol, a strigolactone that does not efficiently induce *S. hermonthica* germination (Karaya et al., 2012). This resistance, qualified as phenotypic resistance, has been identified in other *Striga*‐resistant crop genotypes (Jamil et al., 2011; Robert, 2011). However, resistance associated with low production of Striga seeds germination stimulant may not be related to low production of total strigolactones, but rather to the types of strigolactones released (Yoneyama et al., 2010).

In contrast, postattachment mechanisms act after Striga has attached and attempted to penetrate the host (Figure 4b). These mechanisms result in physiological or biochemical barriers, that prevent Striga haustorium from connecting to the host xylem (van Dam & Bouwmeester, 2016). *Striga hermonthica* postattachment resistance in maize has mainly come from its wild‐grass relatives *Zea diploperennis* (Amusan et al., 2008; Lane et al., 1997) and *Tripsacum dactyloides* (Gutierrez‐Marcos et al., 2003). Post attachment resistance in maize expressed by an incompatibility in ZD05 inbred line with *S. hermonthica* has been observed (Amusan et al., 2008, 2011). In these cases, the parasite penetrated host cortex but was prevented from getting into the host endodermis. The exact mechanism for this parasite's inability to penetrate the endodermis remains unknown. This resistance could be attributed to biochemical or physiological barriers from the host (Amusan et al., 2008; Yoshida & Shirasu, 2009). Recently, postattachment Striga resistance has been shown in the 'KSTP’94', maize open‐pollinated variety (OPV) (Mutinda et al., 2018). However, the molecular mechanisms underlying postattachment Striga resistance are unknown. Preference for OPV is most likely due to the prohibitive price of hybrids or lack of availability of hybrid seed in some SSA countries (Badu‐Apraku & Fakorede, 2017). In addition, these OPV’s are more affordable and consequently easy to multiply and readily available (Midega et al., 2016).

Although hybrids are known and desirable for their high productivity and quality, they have shown reduced pathogen resistance compared to the OPVs which have innate defence traits (Schroeder et al., 2013). It is, therefore, vital to understand the genetic make‐up of the parents used to develop hybrids as this would be more useful for further development of improved maize germplasm with enhanced resistance to *S. hermonthica*.

### 3.2 Potential sources of Striga resistance in maize

Genetic improvement for Striga resistance depends on the availability of germplasm sources with different levels of resistance. Therefore, resistance is prioritized in maize breeding programmes for regions where Striga is endemic and causes major yield losses to farmers. The sources of resistance to Striga have been identified in maize and other crops such as rice, sorghum and cowpea (Amusan et al., 2008; Haussmann et al., 2004; Mbuvi et al., 2017; Menkir, 2006; Yonli et al., 2006) (Table 2).

**TABLE 2:** Potential sources of Striga resistance

Germplasm	Source	Name	Institution	References
Wild‐maize relatives	genes for inhibition of *Striga*haustorial development	*Tripsacum dactyloides, Linea*	IITA	Gurney et al. (2018)
	Resistance	*Zea diploperennis*, Doebley et Guzman		Amusan et al. (2008)
Landraces	horizontal resistance	Broad base	KARI	Midega et al. (2016)
Inbred lines	Resistance/tolerance	TZi 3 (1368 STR), TZi 25 (9450 STR)	IITA	Kim and Akintunde (1989), Konate et al. (2017), Menkir et al. (2006)
		9030, 1393, TESTR151, TESTR 156, OSU231//56/44‐6‐4‐17‐3	CIMMYT KARI	Karaya et al. (2014)
	Resistance	TZill, TZil2, TZi25 TZi30 TZEIOR 108, TZEI 10, TZEI 17	IITA	
		TZISTR1174, TZISTR1162, TZISTR1192	IITA, Uganda National Crop Resources Research Institute,	Simon et al. (2018)
OPV	IITA populations	TZL comp1 synw‐1 and Acr94TZE Comp s‐w	IITA	Menkir and Kling (2007)
	Resistance/tolerance	TZEE‐W Pop STR, TZEE‐Y Pop STR, 2004 TZEE‐Y Pop STR C4, TZEE‐W Pop STR QPM C0 and TZEE‐W Pop STR BC2 C0; TZEE‐W STR 107 BC1, TZEE‐W Pop STR C5, 2012 TZEE‐Y DT STR C5	IITA	Makumbi et al. (2015), Menkir, Franco, et al. (2012), Oyekunle et al. (2017)
	Striga postattachment Resistance	KSTP 94, STR‐VE‐216	KALRO CIMMYT	Mutinda et al. (2018)
Hybrids	Resistant and tolerant	PHB3253, PHB30G19, PHB30B50	Pioneer	Chitagu et al. (2014)
	Resistant and tolerant	MH1416, MQ623, SC643, SC527, SC535	Seed Co Mukushi Seeds	Nyakurwa et al. (2018)
	Resistance/Tolerance	TZISTR1162 × TZISTR1198 TZISTR1199 × TZISTR1181 TZISTR1192 × 1368STR	Uganda National Crop Resources Research Institute	Simon et al. (2018)
	Tolerance	8322‐13, 8321‐18 and 9022‐13, TZEIOR 57 × TZEIOR 108, TZEIOR 57 × TZEIOR 127, TZEIOR 13 × TZEIOR 59, TZEIOR 57 × TZEI 10 and TZEIOR 127 × TZEI 10	IITA	Kim and Akintunde (1989), Konate et al. (2017)

Abbreviations: KALO, Kenya Agricultural Livestock Research Organization (Kakamega, Kenya); KARI, Kenya Agricultural Research Institute.

Striga resistance in maize could be sourced from wild‐grass relatives like *Zea diploperennis* and *Tripsacum dactyloides* (Amusan et al., 2008; Gutierrez‐Marcos et al., 2003; Lane et al., 1997). Such efforts have led to the development of Striga‐resistant inbred line ZD05 suitable for integration in breeding programmes in Western Africa (Kim, 1991). Integrating this breeding line into the breeding programme, IITA in collaboration with National Agricultural Research Systems (NARS) have focused on developing new maize genotypes with the desired trait and adapted to various agro‐ecological regions. Due to Striga proneness in Eastern Africa, maize genotype 'KSTP’94' has been developed and deployed as Striga tolerant source especially in Western Kenya (Mutinda et al., 2018). 'KSTP’94' exhibits remarkable resistance to Striga under field conditions; a characteristic that has made it a subject of intense research in the region as well as in research to understand the mechanism of Striga resistance in maize. Karaya et al. (2012) and Midega et al. (2016), have identified maize landraces that are less affected by *Striga hermonthica* comparatively to hybrids in Western Kenya. These results provide an insight into the potential role of landraces which could play an important role in the efforts towards an integrated management approach for Striga in smallholder cropping systems. The potential genetic variability for *S. hermonthica* resistance can be harnessed from wild‐grass relatives, open pollinated, inbred as well ashybrids lines (Table 2).

Promising Striga resistance genotypes have been identified for further testing and experimental releases in African countries under projects such as Stress Tolerant Maize for Africa (STMA).

### 3.3 Genetics resistance to Striga

Information on the genetic basis of resistance to Striga is critical for plant breeding and selection. Genes action for grain yield and other agronomic traits have been reported for maize under Striga infestation (Ejeta et al., 1997). Resistance evaluation is based on grain yield under Striga infestation, number of Striga plants emerged on the host and host damage syndrome rating. However, there have been contradictory reports on the gene action controlling *Striga* resistance in maize. It is quantitatively inherited with additive gene effects being more important than non‐additive effects. This contributes to regulating the host plant damage syndrome rating and grain yield under *Striga* infestation (Kim, 1994; Berner et al., 995; Akanvou et al., 1997). As reported by Kim (1994) and Berner et al. (1995) different genes control the number of emerged *Striga* plants and the level of host plant damage. Moreover, there is evidence that additive gene action has a higher contribution to natural gene action with regards to grain yield and Striga traits in maize (Akaogu et al., 2013; Badu‐Apraku et al., 2015, 2016; Menkir et al., 2010). In contrast, other studies reported that the impact of non‐additive genes is more important than the effect of additive genes in the control of the inheritance of host plant damage, while the effect of additive genes is more important in the control of the number of emerged *Striga* plants (Gethi & Smith, 2004; Badu‐Apraku et al., 2007; and Yallou et al., 2009). A recent study reported that the dominant effects surpass the additive effects for the number of emerged Striga plants and inheritance of Striga resistance in maize may be conditioned by non‐additive gene action (Akaogu et al., 2019). Additionally, the involvement of epistatic effects in the inheritance of Striga resistance aa in maize has been reported (Adetimirin et al., 2001; Akaogu et al., 2019). Unlike maize, the progress in the identification of genes for marker‐assisted selection in other crops such as sorghum and rice is substantial. The identification of lg gene mutant alleles at the LGS1 (Low Germination Stimulant 1) locus on chromosome 5 of sorghum has reduced significantly the *S. hermonthica* germination stimulant activity (Gobena et al., 2017). This gene was found to code for a sulfo‐ transferase enzyme and when silenced led to a change in 5‐deoxystrigol into orobanchol compounds in the root exudates (Gobena et al., 2017). In addition, other loci have been reported to play important roles in parasitic resistance, including the genes *CCD1*, *CCD7*, *CCD8*, *DAD2*, *MAX1*, *DWARF 53* (D53) and *LBO* (Sun et al., 2008; Hamiaux et al., 2012; Zhou et al. 2013; Aly et al., 2014; Zhang et al, 2014; Brewer et al., 2016). In maize, roots with mycorrhizal formations have shown a higher *ZmCCD1* expression and induced lower germination of Striga (Sun et al., 2008). Evidence for strigolactones and strigolactone perception genes of the MAX‐2‐type in *S. hermonthica*, namely *ShCCD7* and *ShCCD8* has been provided (Liu et al., 2014). In tobacco, the silencing of *CCD7* and *CCD8* genes has delayed the virus parasite formation in the host, indicating that these two genes are a key in the parasitic life cycle (Aly et al., 2014). Recently, some significant loci on chromosomes 9 and 10 of maize that are closely linked to *ZmCCD1* and *amt5* genes, respectively, and may be related to plant defence mechanisms against Striga parasitism have been identified (Adewale et al., 2020).

Availability of all this information on the type of gene action governing the inheritance of resistance to Striga in maize genotypes would, therefore, contribute to the introgression of resistance genes and dissemination of resistant genotypes (Akanvou & Doku, 1998).

## 4 METHODS FOR SCREENING Striga RESISTANCE IN MAIZE

Development of Striga‐resistant cultivars has been limited by the lack of dependable screening techniques (Yagoub et al., 2014). Some of the screening techniques that have been used include field techniques, screen house and laboratory methods (Rodenburg et al., 2015).

Field screening is an artificial technique that consists of uniform infestation with Striga using appropriate experimental design. The procedure of this technique has been described in detail by Badu‐Apraku and Fakorede (2017). Confounding effects of environmental conditions on the polygenic inheritance of traits associated with Striga resistance make field screening indispensable despite the advances made in laboratory and at pot experiments stage.

Screen house technique has been used to screen maize genotypes for tolerance / resistance to Striga (Chitagu et al., 2014; Nyakurwa et al., 2018; Yohannes et al., 2016). In screen houses, screening for varietal resistance has been performed using pots and buried seed studies (Eplee & Norris, 1987; Rao, 1985; Sand et al., 1990). With regard to the pot screening techniques ‘poly bag’ and seed pan, and the ‘Eplee bag’ are used (Eplee, 1992; Rao, 1985). The most important aspect in screen house evaluation is its compatibility with experiments on the efficiency in controlling the Striga vector (Kountche et al., 2019). Several studies have also demonstrated the validity of the Eplee bag technique as a good screening method (Ahonsi et al., 2002; Yonli et al., 2006). Previously, pot experiments were used to access the level of parasite variation in the attachment to the roots of diverse maize inbred lines alongside the plant host interaction (Menkir et al., 2006).

Laboratory methods employed in Striga research have proven to be the best option so far for screening infection. The use of laboratory‐based assays has provided interactive biological processes between Striga and the roots of the host plants during each individual stage of the parasitism process. Hess et al. (1992) developed an in vitro laboratory assay termed such as the agar gel assay (AGA) to determine the genotypic efficacy of host root exudates to germinate preconditioned Striga seeds. This system gave a good correlation with field resistance (Hess et al., 1992; Ramaiah, 1987). These growth systems have been used to examine the architecture of host roots and their biochemical mechanisms of resistance (Amusan et al., 2011; Mohamed et al., 2010; Mrema et al., 2017). Kountche et al. (2019) used AGA to assess the germination‐inducing activity of selected strigolactones (SLs) analogues on *S. hermonthica* seeds. AGA is useful for screening maize genotypes with a high degree of success in identifying Striga*‐*resistant varieties especially those emanating from the wild‐species relatives such as *Z. diploperennis* and *T. dactyloides* (Amusan et al., 2011; Gurney et al., 2003, 2006; Karaya et al., 2012 ). More recently, AGA experiments have been used to determine the levels of resistance or tolerance of new quality protein maize genotypes to *S*. *asiatica* (Nyakurwa et al., 2018).

Furthermore, the rhizotron screening system has been proposed as an ideal technique to circumvent the limits of field technique and initiate a reliable postattachment screening (Rodenburg et al., 2015). Rhizotrons are transparent root observation chambers that enable Striga attached to the host plant to be counted. The AGA technique also allows the evaluation of resistance mechanisms phenotype and determination of the effect of Striga on host biomass over a period of time with minimal disturbance (Rodenburg et al., 2015; Runo et al., 2012). Rhizotron Perspex chambers have been extensively used to screen a variety of host species including maize (Mutinda et al., 2018).

## 5 BREEDING APPROACHES USED FOR Striga RESISTANCE IN MAIZE

Considerable efforts have been made in breeding for Striga resistance in cereals especially in maize and significant progress has been achieved in the development of improved varieties. After the identification of a potential source of resistance, the next critical step in the breeding programme depends on the breeder's ability to incorporate the resistance genes into the best‐adapted varieties. This can be performed with several strategies, amongst which are the conventional and or classical breeding and the marker‐assisted selection (MAS).

### 5.1 Conventional breeding for Striga resistance

Conventional plant breeding aims at increasing the chances of selecting individuals from populations generated from genetic mating designs. Selection has usually been carried out at the whole‐plant level thereby, representing the net result of the interaction between genotype and environment (Badu‐Apraku et al., 2017). However, identification of potential sources of resistance is the first step of all Striga breeding programmes. To access the genes for resistance and incorporate them into well‐adapted varieties, conventional breeding relies on techniques such as recurrent selection, half‐sib or full‐sib selection, S1 family and F1 family (hybrid) selection schemes. Conventional breeding techniques were predominantly used in conferring superior combinations of Striga resistance alleles amongst susceptible cultivars (Menkir et al., 2004). It is, therefore, relevant to explore the applicability of many conventional breeding techniques generally used in various Striga resistance‐breeding programmes.

Recurrent selection is designed to increase the frequency of favourable alleles in a population (Hallauer, 1992; Hallauer & Carena, 2012; Badu‐Apraku & Fakorode, 2017). This procedure has been used effectively in maize to improve quantitatively inherited traits (Badu‐Apraku, 2010; Menkir & Kling, 2007). Few studies have been conducted on the effectiveness of recurrent selection in improving the level of Striga resistance in maize (Menkir & Kling, 2007). Recurrent selection methods capitalize on additive gene action under an effective and reliable artificial method of Striga infestation for the screening of progenies. It facilitates the accumulation of Striga resistance genes to develop germplasm with multigenic resistance that could be sustainable over time and effective for the control of the parasitic weed (Badu‐Apraku et al., 2012; Menkir & Kling, 2007). Recurrent selection has been used successfully to improve grain yield and other agronomic traits in maize populations under infestation (Badu‐Apraku, 2010; Menkir et al., 2004). Through recurrent selection, researchers have reported genetic gains in maize grain yield cultivars under Striga infestation. Menkir et al. (2004) observed that over 2 years selection, Striga damage symptoms were reduced by 3% per cycle, number of emerged Striga plants by 10% per cycle and grain yield increased by 16% per cycle under Striga infestation conditions. Within two periods of selection (1988–2000 and 2001–2006), recurrent selection improved the annual gain yield from 0.86% to 2.11% in early maize under Striga infestation (Badu‐Apraku et al., 2013). This approach has led to an increase in genetic gains in grain yield of 498 kgha^−1^ cycle^−1^ (16.9% cycle^−1^) in 3 years (2014–2017) under Striga infestation (Badu‐Apraku et al., 2019). More recently, genetic gains in maize grain yield and other agronomic traits under Striga condition for periods of selection have been reported. Using recurrent selection, traits associated with grain yield including plant height and the number of ears per plant increased, ear aspect and anthesis–silking interval decreased over time under Striga condition (Menkir & Meseka, 2019). The authors observed that on average, hybrids developed after the 1990s yielded 64% more and displayed 61% less parasite emergence and 30% less parasite damage at 10 weeks after planting compared with hybrids developed before the 1990s.

The half‐sibling selection scheme is also one of the easiest ways in developing composite populations with at least moderate resistance to *S. hermonthica* (John & Sleeper, 1995). The full sib and selection from S1 progeny tests allows for an increased scope of variability in progeny from source populations and greater control over pollen, and should translate into an increased frequency of favourable alleles for Striga resistance in populations under selection (Hallauer, 1992; Menkir et al., 2004).

The backcross breeding procedure is straight forward if a source population or donor, with a high frequency of desirable alleles for Striga resistance is available. Therefore, rapid progress can be achieved in building resistance to Striga if a donor exhibiting high dominance for Striga resistance genes is identified. Under such condition, ideal recurrent parents would be genotypes combining early maturity and high yield (Badu‐Apraku et al., 2017). Germplasm derived through the backcross method forms the basis for cultivar advancements towards achieving polygenic resistance to *S. hermonthica*. Such inbred from *Z. diploperennis* and tropical maize have been essential in the development of *S. hermonthica‐*resistant open‐pollinated populations like *Zea* diplo SYNW‐1, TZL Comp SYNW‐1. Partial resistance to *S. hermonthica* was also observed in backcross hybrids from a resistant donor *T. dactyloides* (Gurney et al., 2018).

Despite the low costs and yield stability benefits associated with the recurrent use of synthetic maize populations, the superiority in performance of hybrid cultivars is being acknowledged with an increasing trend amongst southern African farmers (Badu‐Apraku & Fakorede, 2017). The desire to increase maize yields under marginal growing conditions and a rise in literacy can be the major reasons behind the increase towards the complete adoption of hybrid technology in countries like Zimbabwe, Ghana, Mali and Nigeria (STMA, 2019). Heterosis of hybrid varieties can be useful in mitigating the effect of Striga on maize productivity. With the increased use of hybrid maize seed in West and Central Africa (WCA), Menkir et al. (2004) have selected *S. hermonthica‐*resistant hybrids by crossing diverse inbred lines. These hybrids are able to suppress parasite emergence, with some of them producing high grain yield under high Striga infestation levels (Menkir et al., 2012b). However, multi‐location field screening for Striga resistance resulted in significant genotype × environment (G × E) interactions for Striga resistance traits in maize trials (Akinwale et al., 2014; Nyakurwa et al., 2018; Simon et al., 2018). Based on these results, there is a need to select for specific adaptation in Striga resistance breeding, particularly in the case of contrasting environment where different putative Striga ecotypes may exist.

### 5.2 Marker‐assisted breeding for Striga resistance

Marker‐assisted selection (MAS) is an indirect selection process where a trait of interest is selected based on a marker linked to the trait, rather than on the trait itself (Ribaut et al., 2001). This breeding method allows the performance of a selected phenotype to be predicted based on the use of molecular markers at early generation.

Application of molecular markers has provided significant opportunities for breeders to characterize, evaluate and select maize germplasm widely used by public and private sectors. Molecular markers are also used for screening crop genotypes for tolerance to biotic or abiotic stress. Using SSRs and SNPs markers, some elite genotypes for the breeding of Striga resistance are selected and new makers have been identified, which significantly contributed to the differentiation of Striga tolerant and susceptible genotypes (Bawa et al., 2015; Shayanowako et al., 2018). Molecular markers can better help in the assessment of relatedness in isogenic lines to determine families that can be bulked or discarded, which in turn can reduce maintenance costs (Dean et al., 1999).

Several researchers have reported the efficiency and superiority of MAS and its effective integration into mainstream maize breeding programmes. Efforts deployed with the use of molecular tools can be utilized in determining families that can be bulked or discarded. Those families could also help in the selection of parental lines for Striga‐resistant hybrids development with high yields and stable across many agroecologies (Akinwale et al., 2014; Mengesha et al., 2017).

Molecular marker technologies and the construction of genetic linkage maps have made it possible to detect genetic loci associated with complex traits (Kang et al., 1998; Sibov et al., 2003). Genetic linkage maps and quantitative trait loci (QTL) mapping technology have enhanced the efficiency of estimating the number of loci controlling genetic variation in a segregating population and the characterization of the map positions in the genome (Xiao et al., 1996). In maize, QTLs identification was focused mainly on abiotic and biotic stresses such as drought tolerance (Semagn et al., 2015; Tuberosa et al., 2002), low soil nitrogen (Mandolino et al., 2018; Ribeiro et al., 2018), pests (Jiménez‐Galindo et al., 2017) and foliar diseases (Gowda et al., 2018). In SSA, little progress has been reported on the detection of QTLs or genes for Striga resistance in maize. However, QTLs for resistance to *S. hermonthica* have been identified from local populations including wild relatives and successfully transferred through backcross breeding into adaptable maize populations (Rich & Ejeta, 2008). Using the linkage mapping method, two putative QTLs have been discovered that govern incompatible response to Striga parasitism in maize amongst F2 segregated populations (Amusan, 2010). Whereas some QTLs have been discovered for Striga resistance in sorghum and rice (Atera et al., 2015; Yasir & Abdalla, 2013; Yohannes et al., 2015; Ali et al., 2016). Using genomic association wide (GWA), 24 SNPS markers associated with grain yield, Striga damage at 8 and 10 weeks after planting (WAP), ears per plant and ear aspect under Striga infestation were detected in early maturing maize inbred (Adewale et al., 2020). Therefore, there is an urgent need to identify QTLs for Striga resistance to facilitate the rapid and efficient transfer of the genes into other maize genotypes.

## 6 WAY FORWARD ON Striga RESISTANCE IN MAIZE AND CONCLUSION

Breeding maize for Striga resistance is challenging due to the scarcity of resistant sources in cultivated species. In this review, we explored the integrated approach using resistant cultivars is the most effective option, since Striga‐resistant cultivars play a major role in reducing Striga pressure, both in terms of Striga count and vigour compared with individual control options. In general, many breeding techniques are used in maize breeding programmes for Striga resistance. However, conventional breeding techniques through the screening of resistant genotypes are the most frequently used in the maize breeding programmes in Africa. Screening of resistant genotypes under artificial Striga infestation is very expensive, time‐consuming and labour intensive. Moreover, obtained results are often not consistent due to genotype by environment interactions, inability to assess evenness of Striga distribution and ascertain contact between Striga and host roots.

Another possibility is to develop high yielding maize genotypes with resistance to Striga using genome editing of SLs genes, which are responsible for Striga germination and attachment. It might be a direct way of increasing maize grain yield in Striga endemic locations of SSA. At present, accumulation of resistance QTLs in most programmes may be facilitated by conventional breeding techniques and the use of cost‐effective molecular markers (Badu‐Apraku & Fakorede, 2017). The present challenge is to convert a large amount of available genetic information into a large set of markers useful for Striga resistance breeding in maize and to integrate such markers into a sustainable breeding scheme. Further exploration of closely related QTL gene markers related to Striga will help in the effective trait pyramiding gene actions that can contribute to maize effective production. Some effective molecular docking approaches such as CRISPR/Cas9 genome editing of strigolactone genes, which are responsible for *Striga* germination and attachment could also be considered for the development of high yielding maize genotypes with resistance to Striga.

## CONFLICT OF INTERESTS

The authors declare that there is no conflict of interests.

## AUTHOR CONTRIBUTIONS

A.M. Yacoubou, wrote the draft manuscript with the contributions of all the co‐authors.
